# Disturbed Spatial Learning and Hippocampal Molecular and Histological Alterations in Middle-Aged Rats with a History of Polycystic Ovary Syndrome

**DOI:** 10.5812/ijem-167083

**Published:** 2026-08-31

**Authors:** Mahsa Noroozzadeh, Mostafa Changaei, Mahbanoo Farhadi-Azar, Maryam Mousavi, Pouria Mousapour, Mina Amiri, Masoumeh Jorjani, Fahimeh Ramezani Tehrani

**Affiliations:** 1 Reproductive Endocrinology Research Center, Research Institute for Endocrine Molecular Biology, Research Institute for Endocrine Sciences, Shahid Beheshti University of Medical Sciences Tehran Iran; 2 Department of Immunology, Faculty of Medical Sciences, Tarbiat Modares University Tehran Iran; 3 Department of Midwifery and Reproductive Health, School of Nursing and Midwifery, Tehran University of Medical Sciences Tehran Iran; 4 Foundation for Research and Education Excellence Vestavia Hills, AL USA; 5 Department of Pharmacology, Neurobiology Research Center, School of Medicine, Shahid Beheshti University of Medical Sciences Tehran Iran

**Keywords:** Polycystic Ovary Syndrome, Learning, Memory, Hippocampus, Rat

## Abstract

**Background:**

Studies have reported inconsistent findings regarding the potential effects of polycystic ovary syndrome (PCOS) on neurocognitive function.

**Objectives:**

This study aimed to investigate whether cognitive function, particularly spatial learning and memory, and hippocampal tissue are impaired in middle-aged rats with a history of PCOS.

**Methods:**

Spatial learning and memory, hippocampal histopathology, and the expression of genes including beta-secretase 1 (bace1), microtubule-associated protein tau (mapt), amyloid-beta precursor protein (app), nestin (nes), and doublecortin (dcx) in hippocampal tissue were compared between middle-aged PCOS rats and controls (n = 7/group).

**Results:**

During training (learning), middle-aged PCOS rats had significantly higher mean latency (22.04 (1.86, 42.22); P = 0.03) and duration (5.62 (1.41, 9.84); P = 0.009) than controls. However, memory performance did not differ significantly between PCOS rats and controls. PCOS rats had a significantly higher number of damaged neurons in the CA1 (24.25 ± 2.68 vs. 9.50 ± 0.64), CA2 (20.75 ± 1.03 vs. 8.25 ± 1.10), CA3 (20.50 ± 1.65 vs. 8.50 ± 1.04), and DG (28.25 ± 1.88 vs. 13.00 ± 1.29) regions of the hippocampus than controls (P < 0.05), and they showed mild hippocampal damage. In PCOS rats, bace1 gene expression was significantly increased in hippocampal tissue, whereas mapt gene expression was decreased compared with controls. The expression levels of the other genes did not differ significantly between the two groups.

**Conclusions:**

A history of PCOS during reproductive age may increase the risk of cognitive dysfunction, particularly impaired spatial learning, as well as hippocampal damage later in life.

## 1. Background

Alzheimer disease (AD) is characterized by progressive deterioration of cognitive function, typically emerging in middle or late adulthood, and is accompanied by heterogeneous behavioral symptoms. AD is among the most common neurodegenerative disorders and is a major cause of dementia in older adults (1).

Polycystic ovary syndrome (PCOS), the most common endocrinopathy in women of reproductive age, is characterized by androgen excess, ovulatory dysfunction, and polycystic ovarian morphology. Conditions associated with PCOS, such as insulin resistance (IR), type 2 diabetes mellitus (T2DM), obesity, inflammation, gut microbiota (GM) dysbiosis, depression, and sleep disturbances (2-4), may adversely affect brain structure and cognitive function (5-8), potentially increasing the risk of cognitive impairment and AD later in life.

Despite several animal and human studies examining the possible effects of PCOS on neurocognitive function (9-19), evidence regarding its impact on brain health remains limited. Further studies are required to clarify the neurological and cognitive consequences of PCOS, its potential association with AD, and the underlying mechanisms.

Human studies in this field face several challenges, including loss to follow-up in long-term studies, ethical constraints, and limited feasibility of mechanistic investigations. Therefore, validated animal models of PCOS allow controlled investigation of the syndrome’s cognitive and neuronal implications. We previously established a validated rat model of PCOS that reproduces key reproductive and metabolic features of the human condition (20-22).

## 2. Objectives

In this study, we aimed to examine spatial learning and memory performance (cognitive function), hippocampal tissue, and the expression of genes involved in AD pathogenesis and neural repair and proliferation, including Beta-secretase 1 (bace1), microtubule-associated protein tau (mapt), amyloid-beta precursor protein (app), Nestin (nes), and doublecortin (dcx) (23-29), in the hippocampus of middle-aged rats (12 months of age) (30, 31) with a history of PCOS (middle-aged PCOS rats) compared with control rats (non-PCOS rats).

## 3. Methods

### 3.1. Animals and Experimental Design

Ten female Wistar rats (85 - 90 days old, 185 ± 10 g) were obtained from the Research Institute for Endocrine Sciences (RIES) animal facility. One male and one female rat were housed together overnight in a polypropylene cage under standard conditions (12-hour light/dark cycle, temperature 22 ± 3°C, and relative humidity 45 - 55%). The presence of sperm on vaginal cytology was considered day 1 of pregnancy. Pregnant rats were randomly allocated to the experimental and control groups (n = 5/group).

The rat model of PCOS was generated according to our previously published method (20). Pregnant rats in the experimental group received a subcutaneous injection of 5 mg free testosterone, whereas pregnant rats in the control group received only the solvent (sesame oil and benzyl benzoate). After weaning, female offspring were maintained under standard conditions with ad libitum access to food and water. Prenatal testosterone-exposed female offspring were considered the rat model of PCOS (n = 7), and non-exposed rats served as controls (n = 7). PCOS rats and controls were followed up to 12 months of age and assessed for spatial learning and memory performance, hippocampal histopathology, and the expression of genes involved in AD pathogenesis (bace1, mapt, app, nes, and dcx) in hippocampal tissue. A summary of the experimental design is shown in Figure 1.

**Figure 1.**
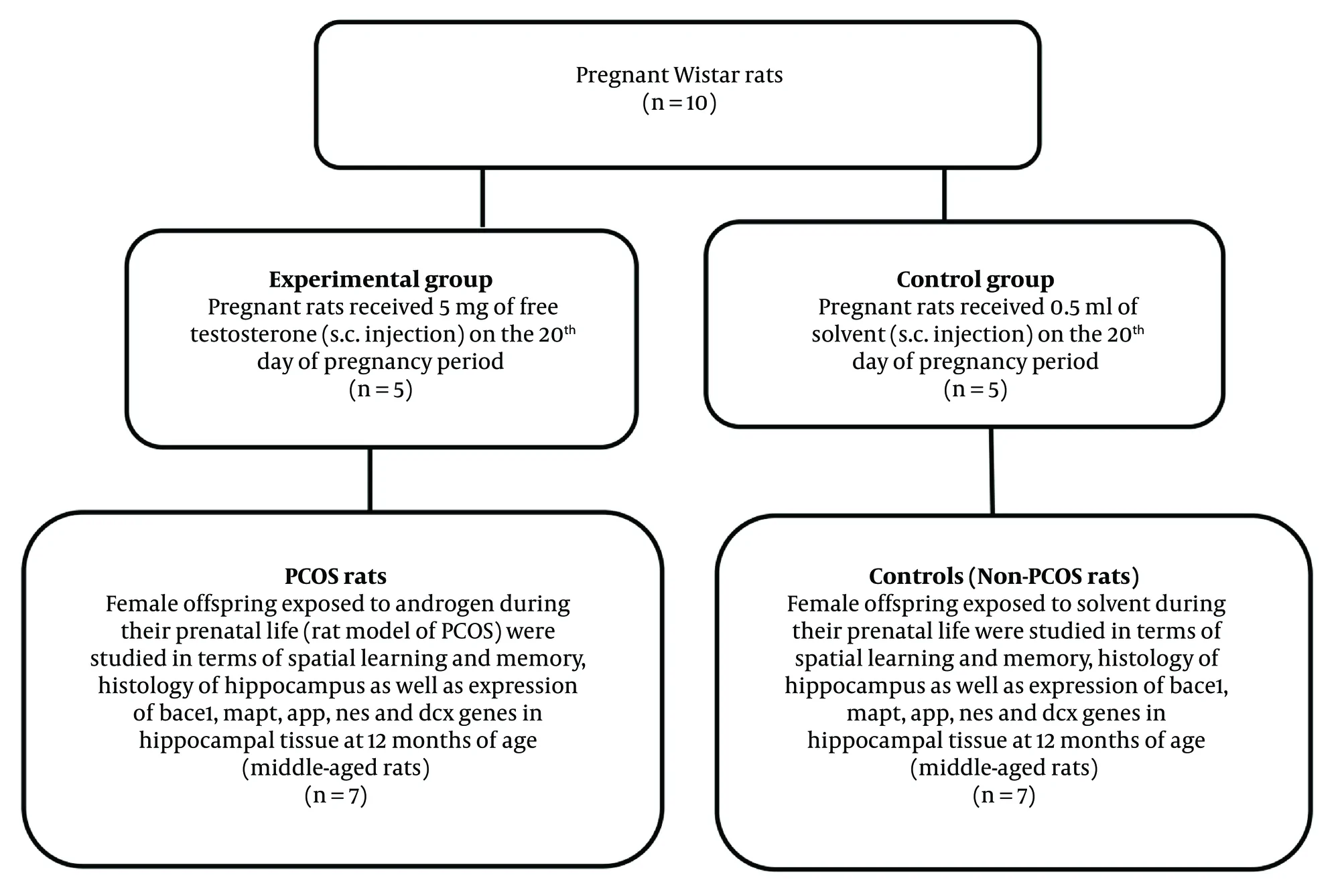
Study flowchart. Solvent sesame oil and benzyl benzoate at a ratio of 4:1. Abbreviations: s.c., subcutaneous; PCOS, polycystic ovary syndrome; bace1, beta-secretase 1; mapt, microtubule associated protein tau; app, amyloid-beta precursor protein; nes, nestin; dcx, doublecortin

### 3.2. Reconfirmation of the Rat Model of PCOS

Key PCOS-related characteristics, including elevated serum testosterone, disrupted estrous cyclicity, and increased anogenital distance (AGD), were assessed in prenatally androgenized rats during reproductive age (3 - 4 months) and compared with control animals (20, 21); the findings confirmed that the rat model of PCOS had been appropriately generated and validated (data not shown).

### 3.3. Measurement of Body Weights in PCOS and Control Rats

Body weights (BWs) of PCOS rats and controls were measured at birth (day 0) and at 15, 30, 45, 60, 75, 90, 120, 180, 270, and 360 days of age. BW was measured using a scale (Sartorius, Gottingen, Germany) with 1 g accuracy.

### 3.4. Morris Water Maze Test

Spatial learning and memory performance were evaluated in middle-aged PCOS and control rats using the Morris water maze (MWM) over a five-day testing period (32). Latency to escape, time spent in the target quadrant (duration), and platform-crossing frequency were quantified using video-tracking software for subsequent behavioral analysis.

### 3.5. Habituation

Twenty-four hours before the training protocol, each rat was placed in the pool without a platform for 60 seconds (33). This habituation stage facilitated acclimatization to the pool environment and reduced the effect of stress associated with initial exposure on performance.

### 3.6. Reference Spatial Learning and Memory

The training phases were conducted over three consecutive days (days 1, 2, and 3), with each day consisting of four trials; an interval of 10 - 20 min was maintained between trials. In each trial, the rat was released from a random starting point and allowed to swim for up to 60 seconds to find the hidden platform, and the rat’s behavior was recorded within 60 s. Upon finding the platform, the rat was allowed to remain on it for 20 seconds before the next trial. Rats unable to find the platform within 60 seconds were guided by the examiner to the platform and allowed to remain on it for 20 seconds. During the training sessions, latency to reach the platform, duration, and platform-crossing frequency were reported as measures of spatial learning. Twenty-four hours after the three-day training phase, the platform was removed, and the rat was allowed to swim in the pool for 60 seconds. A probe trial was performed to assess the memory performance of the study rats. On the probe day, latency to the first platform location, duration, and the frequency of crossings over the platform location were reported.

### 3.7. Histological Assessments

After induction of deep anesthesia with pentobarbital sodium (60 mg/kg body weight), animals from both groups were euthanized by cardiac puncture, and the brains were harvested (n = 4 rats per group) and immediately fixed in 10% formalin for 7 - 10 days at 4°C. Brain tissues underwent standard dehydration procedures followed by paraffin embedding for histological preparation. Coronal hippocampal sections, 7 μm thick, were obtained and processed for Nissl staining. Tissue sections were mounted on glass slides, deparaffinized in xylene, rehydrated through a graded ethanol series followed by distilled water, and then stained with 0.1% cresyl violet solution (Nissl staining). After rinsing with distilled water, sections were dehydrated through ascending ethanol concentrations, cleared in xylene, and mounted with cover slips. Manual quantification of neurons was conducted across distinct hippocampal subfields, including Cornu Ammonis areas 1, 2, and 3 (CA1, CA2, CA3) and the dentate gyrus (DG), within a defined plane range based on the stereotaxic coordinates of Paxinos and Watson; an area of 0.1 mm^2^ was covered. Neuron counts were performed in five histological sections (magnification 100×) across hippocampal regions using Image-J software.

Histopathological assessment defined damaged neurons as those showing loss of membrane integrity and absence of a visible, well-defined nucleus (structural damage). Specimens were examined at 400× magnification; structures with ambiguous morphology were excluded from the analysis to ensure accuracy. Hippocampal damage in the DG, CA1, CA2, and CA3 regions was graded based on the percentage of damaged neurons: Grade 0 (normal), Grade 1 (minimal; < 25%), Grade 2 (mild; 25 - 50%), Grade 3 (moderate to severe; 50 - 75%), and Grade 4 (massive; > 75%) (34). All slides were evaluated in duplicate by investigators blinded to the experimental conditions (35).

### 3.8. Molecular Assessment

RNA was extracted from hippocampal tissue using the TRIzol reagent protocol. RNA concentration and purity were assessed using the 260/280 nm absorbance ratio with a NanoDrop spectrophotometer (Thermo Fisher). To produce complementary DNA (cDNA), total RNA was first treated with DNase I (Thermo Fisher Scientific, USA) to remove genomic DNA contamination. Subsequently, cDNA was synthesized from total RNA using a cDNA synthesis kit (4500 YT, Yekta Tajhiz, Iran) via reverse transcription with random hexamers and an initial oligo-dT primer, according to the manufacturer’s protocol. Gene expression in hippocampal tissue was assessed using quantitative real-time PCR (qRT-PCR) with SYBR Green and gene-specific primers, with sequences provided in Table 1.

**Table 1.** Sequence of Primers ^a^

Genes and Directions	Sequence
**bace1**	
F	ACTGGCAGTCTCTGGTACAC
R	ACTGTCCACGATGCTCTTGTC
**mapt**	
F	CCTGACCTTGCATGACCTCC
R	CTCCTTCTACCGCATCGTCC
**app**	
F	GAGGACTGACCACTCGACCA
R	TCATGTCCGAACTCCGCATC
**nes**	
F	GGTGACAGAGCGAGATGAGG
R	CTGGCAAAATGCCTTGGGTC
**dcx**	
F	CTGTTCATGATCCAGGACTGTCT
R	CCAGGGATTCTGAACTCTGTTGA
**ywhaz**	
F	AGGCAGAGCGATACGATGAC
R	CGAGACGACCCTCCAAGATG

^a^ Abbreviations: F, forward primer; R, reverse primer; bace1, beta-secretase 1; mapt, microtubule-associated protein tau; app, amyloid-beta precursor protein; nes, nestin; dcx, doublecortin; ywhaz, tyrosine 3-monooxygenase. Gene duplication was performed with applied Biosystem and SYBR-Green master mix (Ampliqon).

Melting curve analysis was performed to confirm amplification specificity and exclude non-specific products or primer-dimer formation. Melting curve analysis verified that no primer-dimer formation or non-specific amplification products were present in the assay. Cycle threshold (CT) values were measured. The 2^−ΔΔCt^ method was used to quantify relative gene expression levels, with ywhaz as the internal reference gene. Gene expression levels were analyzed using the Livak method to determine relative fold changes. Three candidate reference genes (b2m, ywhaz, and hprt1) were initially evaluated for normalization. Expression stability was assessed by comparing CT values across all samples and experimental groups. ywhaz showed the lowest CT variability and the most consistent expression pattern and was therefore selected as the endogenous reference gene for normalization of target gene expression.

PCR amplification was performed using the Rotor-Gene 6000 Real-Time PCR system (Corbett Research, Sydney, Australia) with a 40-cycle protocol. The thermal protocol began with an initial denaturation at 95°C for 10 minutes, followed by cycles of denaturation at 95°C for 10 seconds, annealing at 58°C for 10 seconds, and extension at 70°C for 20 seconds. Amplification was quantified by tracking fluorescence intensity in real time.

### 3.9. Statistical Analysis

A generalized estimating equation (GEE) model was applied to assess changes in BWs with increasing age and to evaluate longitudinal trends over time in latency to reach the platform, time spent in the target quadrant, and frequency of platform crossings in both rat groups. The GEE model accounts for correlations among repeated measures within subjects by using a specified working correlation matrix. This framework was used to characterize longitudinal changes in spatial learning across the study groups and can accommodate incomplete repeated-measures data.

Time-trend models were fitted separately by PCOS status, and marginal means and associated p values were reported. Independent-samples t tests were used to compare latency to the first platform location, duration, and platform-crossing frequency on the probe-trial day, histopathological measures, and BWs between groups. The Mann-Whitney test was used to compare molecular assessment results between middle-aged PCOS rats and controls.

STATA software (version 12; College Station, TX, USA) was used for statistical analysis. A P value < 0.05 was considered statistically significant.

## 4. Results

### 4.1. Body Weights

As shown in Figure 2, BWs were compared between PCOS rats and controls at different ages. BWs in PCOS rats at 15 (25 ± 1.69 vs. 21 ± 0.30 g; P = 0.03), 30 (62.57 ± 3.41 vs. 45 ± 1.52 g; P = 0.001), 60 (154.42 ± 3.02 vs. 140.14 ± 4.5; P = 0.02), 75 (173.57 ± 2.95 vs. 159.14 ± 4.13; P = 0.01), and 90 (189.71 ± 2.79 vs. 181.71 ± 1.79; P = 0.03) days of age were significantly higher than those in controls. No significant differences were observed between the two groups at birth (day 0) (6.38 ± 0.30 vs. 6.03 ± 0.17 g; P = 0.33), 45 (110.14 ± 4.02 vs. 105.42 ± 2.74 g; P = 0.35), 120 (200.28 ± 5.14 vs. 199.85 ± 2.91; P = 0.94), 180 (218.57 ± 4.11 vs. 216.28 ± 3.55; P = 0.68), 270 (227.71 ± 5.43 vs. 223.71 ± 3.27; P = 0.54), and 360 (233.57 ± 4.03 vs. 238 ± 3; P = 0.39) days of age.

**Figure 2.**
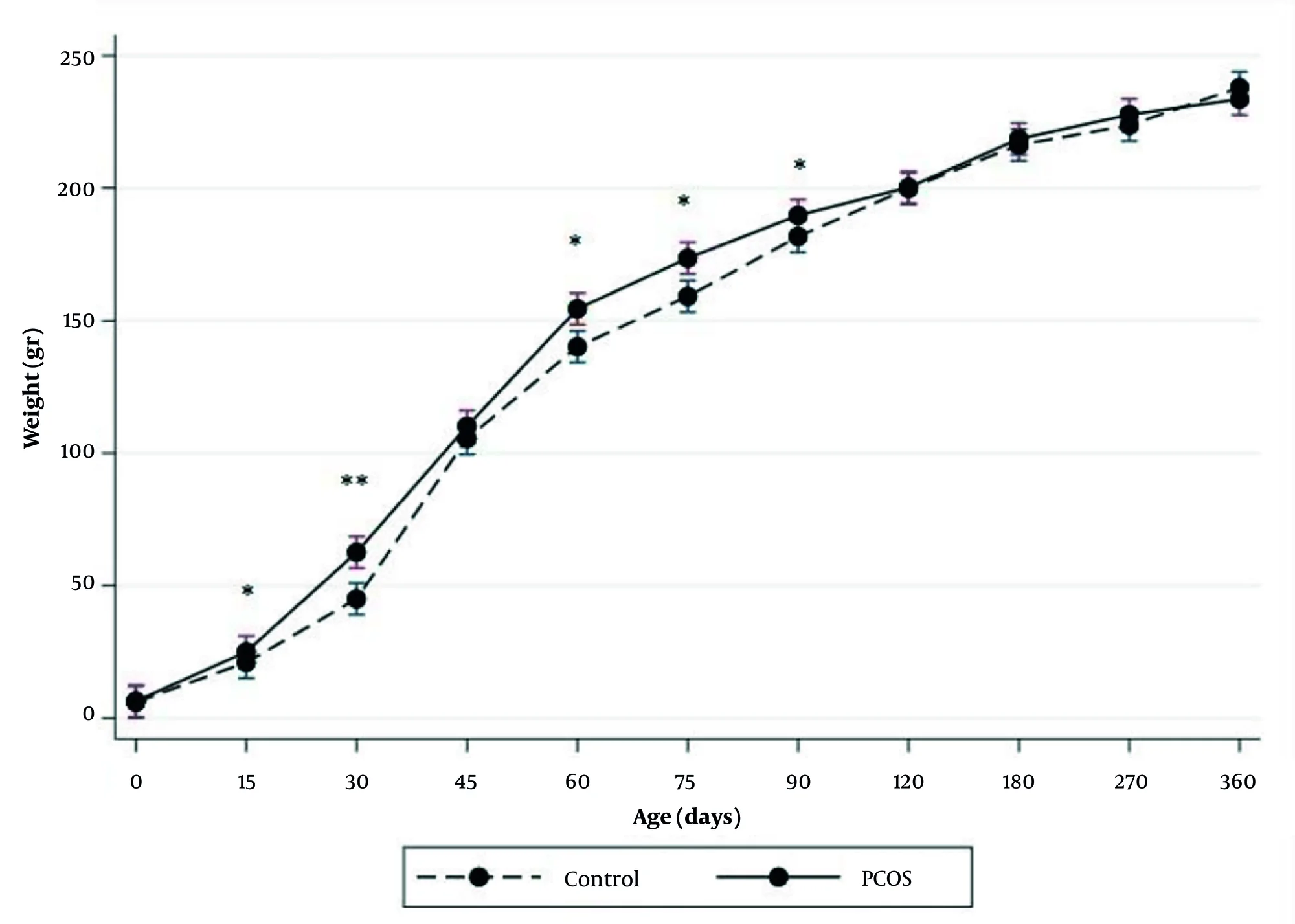
Comparison of mean body weight (BW) in middle-aged PCOS rats (n = 7) and controls (n = 7) at different ages. Values are expressed as means (SE). An independent Student’s t-test was used to compare the results between the two groups and generalized estimating equation (GEE) model was employed to assess the changes of BWs with increasing age. * P < 0.05, ** P < 0.01. Abbreviation: PCOS, polycystic ovary syndrome.

### 4.2. MWM Test

#### 4.2.1. Learning

Table 2 and Figure 3 present the GEE results for latency, duration, and platform-crossing frequency by PCOS status during training days 1, 2, and 3. Mean latency (22.04 (1.86, 42.22) (P = 0.03)) and mean duration (5.62 (1.41, 9.84) (P = 0.009)) were significantly higher in middle-aged PCOS rats than in controls. Mean platform-crossing frequency did not differ significantly (7.11 (-12.42, 26.65) (P = 0.47)) between middle-aged PCOS rats and controls.

**Table 2.** Estimation of the Generalized Estimating Equation (GEE) Model of Latency, Duration, and Frequency in Middle-Aged PCOS Rats vs Controls Over Training Days (Days 1, 2, and 3) ^a^

Variables and Terms	Coefficient (95% CI)	P-Value
**Latency to the platform (sec)**		
PCOS	22.04 (1.86, 42.22)	0.03
Time	-8.39 (-14.42, -2.36)	0.006
Time × PCOS	-7.91 (-16.72, 0.90)	0.08
**Duration (sec)**		
PCOS	5.62 (1.41, 9.84)	0.009
Time	-1.51 (-2.71, -0.30)	0.01
Time × PCOS	-2.16 (-3.88, -0.45)	0.01
**Platform crossing frequency**		
PCOS	7.11 (-12.42, 26.65)	0.47
Time	0.46 (-5.34, 6.28)	0.87
Time × PCOS	-1.16 (-9.69, 7.37)	0.79

^a^ Training Day 1, 2, and 3 represent learning. Abbreviation: PCOS, polycystic ovary syndrome.

**Figure 3.**
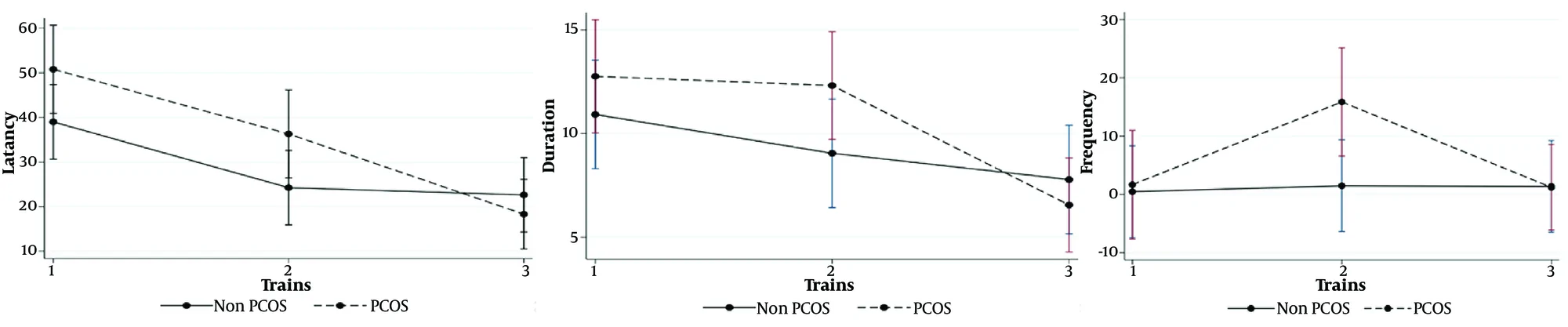
Generalized estimating equation (GEE) measures. Mean changes of latency, duration, and frequency in different days of training (days 1, 2, and 3) between middle-aged PCOS rats and controls (non-PCOS), assuming the interaction between time and study group (n = 7 in each group). PCOS, polycystic ovary syndrome; Training days 1, 2, and 3 learning.

#### 4.2.2. Memory

Table 3 presents independent-samples t-test comparisons of mean latency (26.22 ± 8.12 vs. 17.61 ± 6.52) (P = 0.43), mean duration (20.58 ± 1 vs. 22.69 ± 0.65) (P = 0.13), and mean platform-crossing frequency (1.71 ± 0.42 vs. 2.42 ± 0.71) (P = 0.4) between middle-aged PCOS rats and controls on the probe-trial day. Accordingly, no significant differences were observed between the two groups in mean latency, time spent in the target quadrant, or platform-crossing frequency on the probe-trial day.

**Table 3.** Comparison of Latency, Duration, and Frequency Between Middle-Aged PCOS Rats and Controls on the Probe-Trial Day ^a^

Variables	Controls	PCOS	P-Value
**Latency to the first platform location (sec)**	17.61 ± 6.52	26.22 ± 8.12	0.43
**Duration (sec)**	22.69 ± 0.65	20.58 ± 1.00	0.13
**Frequency of crossing the platform location**	2.42 ± 0.71	1.71 ± 0.42	0.40

^a^ Values are expressed as mean ± SE. Probe-trial day represents memory. Independent-samples t tests were used to compare the groups. Abbreviation: PCOS, polycystic ovary syndrome.

### 4.3. Histopathological Assessment

Findings from hippocampal Nissl staining are shown in Table 4 and Figure 4. Independent-samples t tests indicated a significant increase in the number of damaged neurons in the CA1 (24.25 ± 2.68 vs. 9.50 ± 0.64 (P = 0.01)), CA2 (20.75 ± 1.03 vs. 8.25 ± 1.10 (P < 0.001)), CA3 (20.50 ± 1.65 vs. 8.50 ± 1.04 (P = 0.001)), and DG (28.25 ± 1.88 vs. 13.00 ± 1.29 (P = 0.001)) regions in middle-aged PCOS rats compared with controls, respectively. Grading of hippocampal damage and the percentage of damaged neurons across the DG, CA1, CA2, and CA3 regions in middle-aged PCOS rats and control animals are presented in Table 5. The results indicate that middle-aged PCOS rats exhibited mild hippocampal damage, whereas the control group showed only minimal histopathological alterations.

**Table 4.** Comparison of the Number of Damaged Neurons in Middle-Aged PCOS Rats and Controls ^a^

Hippocampal Region	Controls	PCOS	P Value
**DG**	13.00 ± 1.29	28.25 ± 1.88	0.001
**CA1**	9.50 ± 0.64	24.25 ± 2.68	0.01
**CA2**	8.25 ± 1.10	20.75 ± 1.03	< 0.001
**CA3**	8.50 ± 1.04	20.50 ± 1.65	0.001

^a^ Values are expressed as mean ± SE. Independent-samples t tests were used to compare the groups. Abbreviations: PCOS, polycystic ovary syndrome; DG, dentate gyrus; CA1, CA2, CA3, cornu ammonis 1, 2, and 3.

**Table 5.** Histopathological Grading and Percentage of Damaged Neurons in the Hippocampus of Middle-Aged PCOS and Control Rats ^a^

Hippocampal region	Controls (% Damaged Neurons)	Grade (score)	PCOS (% Damaged Neurons)	Grade (Score)
**DG**	16.32	1	40.63	2
**CA1**	14.39	1	35.92	2
**CA2**	7.73	1	29.74	2
**CA3**	10.07	1	31.37	2

^a^ Grade 0, no damaged cells (normal); Grade 1, damaged neurons < 25% (minimal damage); Grade 2, damaged neurons 25 - 50% (mild damage); Grade 3, damaged neurons 50 - 75% (moderate/severe damage); Grade 4, damaged neurons > 75% (massive damage). Abbreviations: PCOS, polycystic ovary syndrome; DG, dentate gyrus; CA1, CA2, CA3, cornu ammonis 1, 2, and 3.

**Figure 4.**
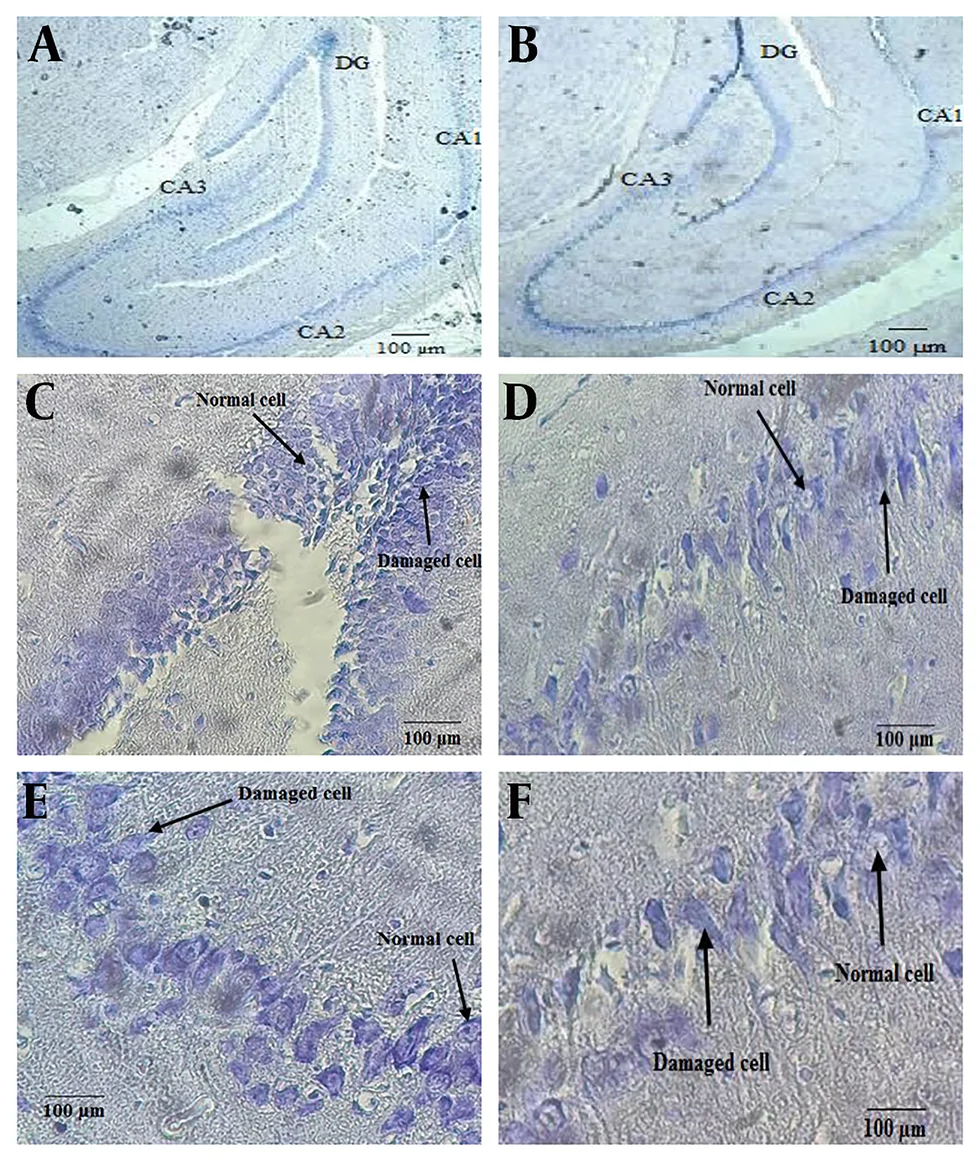
Histology of rat hippocampal tissue. Nissl staining, A, hippocampus in control rats; and B, hippocampus in middle-aged PCOS rats, with 40× magnification. C, DG region of hippocampus, D, CA3 region of hippocampus, E, CA2 region of hippocampus, F, CA1 region of hippocampus with 400× magnifications. Abbreviations: DG, dentate gyrus; CA1, cornu ammonis 1, CA2, cornu ammonis 2, CA3, cornu ammonis 3; PCOS, polycystic ovary syndrome.

### 4.4. Molecular Assessment

Relative bace1 expression in hippocampal tissue from middle-aged PCOS rats was significantly higher than that in controls (24.70 (7.88 - 31.25) vs. 0.99 (0.79 - 1.3)) (P = 0.015) (Figure 5). In contrast, relative mapt expression in hippocampal tissue from middle - aged PCOS rats was significantly lower than that in controls (0.23 (0.13 - 0.31) vs. 1.21 (0.54 - 1.66)) (P = 0.015) (Figure 5). No significant differences were detected in the expression of app (0.29 (0.05 - 0.69) vs. 1.25 (0.62 - 1.4)) (P = 0.114), nes (0.30 (0.17 - 0.65) vs. 1 (0.38 - 4.01)) (P = 0.2), and dcx (0.26 (0.13 - 0.47) vs. 0.93 (0.46 - 2.62)) (P = 0.057) in middle - aged PCOS rats compared with controls (Figure 5).

**Figure 5.**
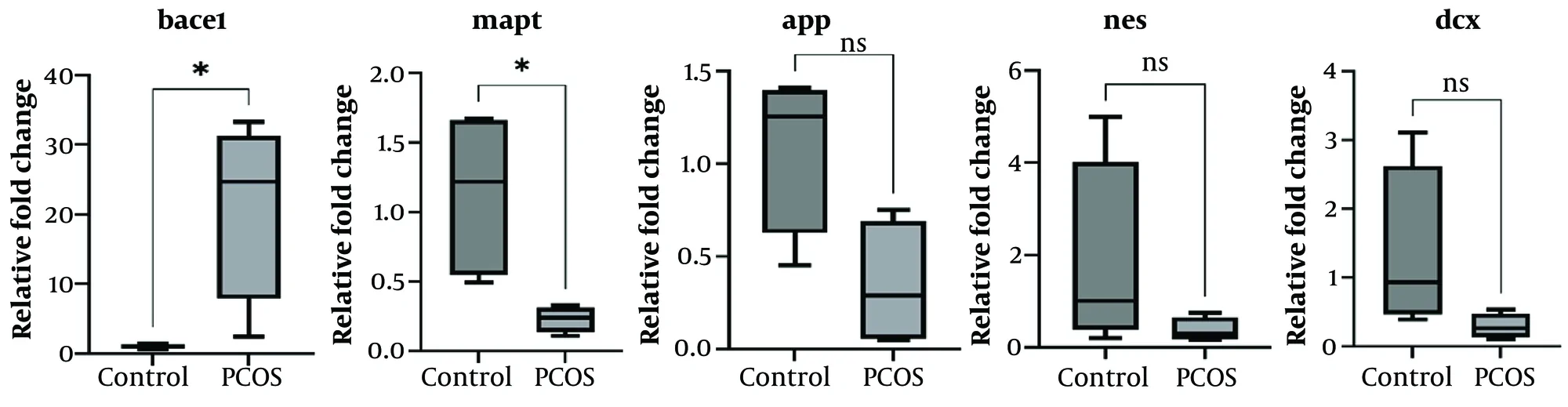
Gene expression. Expression levels of bace1 (Beta-secretase 1), mapt (Microtubule associated protein tau), app (Amyloid-beta precursor protein), nes (Nestin), dcx (Doublecortin) genes (mRNA) in hippocampal tissue in middle-aged PCOS rats and controls. Data are presented as median (interquartile), the number of animals in each group is 7. Mann-Whitney test was used for comparing the results between two groups (middle-aged PCOS rats and controls). PCOS, polycystic ovary syndrome, *P < 0.05. Abbreviation: ns, non-significant.

## 5. Discussion

In the present study, we observed impaired spatial learning, indicative of cognitive dysfunction, along with neuronal damage in the hippocampal tissue of middle-aged PCOS rats. Additionally, in the hippocampal tissue of middle-aged PCOS rats, bace1 gene expression was increased, whereas mapt gene expression was decreased. However, no statistically significant differences were observed in memory performance or in the expression of app, nes, and dcx genes when comparing middle-aged PCOS rats with their controls.

The hippocampus plays a crucial role in spatial learning and memory performance, and its dysfunction is linked to the cognitive impairment observed in neurodegenerative diseases such as AD. In the initial stages of AD, the hippocampal tissue and its connections with other brain regions become damaged (36). In the present study, impaired spatial learning in middle-aged PCOS rats may be due to changes in hippocampal tissue, including neuronal damage. The cognitive dysfunction and hippocampal tissue damage observed in our rat model of PCOS are consistent with findings previously reported in both animal models and women with PCOS, as well as in animals with hormonal disorders (9-14, 37-39). In one previous study, neuronal degeneration in the hippocampus was observed in PCOS rats (9). Neurocognitive decline in a letrozole-induced rat model of PCOS was also reported (10). Another study showed necrotic or degenerative hippocampal pyramidal cells in PCOS rats (37). Additionally, impaired cognitive function and decreased hippocampal volumes were observed in women with PCOS (11, 40).

Neuronal damage and alterations in gene expression in the hippocampal tissue, as well as impaired spatial learning, were observed in our middle-aged PCOS rats. Consistent with findings from both human and animal research, hippocampal impairment and the accompanying deficits in cognitive performance, particularly impaired spatial learning, are likely influenced by several interconnected mechanisms. First, these changes may arise from the direct effects of prenatal androgen exposure (PAE) on hippocampal tissue. PAE disrupts neurodevelopment in critical brain regions, such as the hippocampus, leading to neurobehavioral alterations in offspring (41, 42). Evidence suggests that androgenic signaling is essential for regulating hippocampal neurogenesis and the survival of neurons and glia in offspring (43). Consequently, PAE is associated with impaired neurogenesis and neuronal survival, decreased neuron numbers, impaired density and maturation in the hippocampus, as well as overexpression of Nr4a2 (a crucial molecule involved in offspring impairment), leading to neurobehavioral abnormalities in animals and humans later in life (41, 43). These neurodevelopmental impairments due to PAE are driven by multifaceted pathways, including epigenetic modifications (e.g., DNA methylation and histone acetylation), oxidative stress, and dysregulated hormonal and gene expression patterns, which collectively compromise the structural and functional integrity of circuits involved in emotion and cognition, including the hippocampus (42).

Alternatively, such alterations may result from indirect pathways, whereby PAE induces systemic metabolic and physiological disturbances. Specifically, this exposure is associated with subsequent conditions such as hyperandrogenemia, GM dysbiosis, insulin resistance (IR) due to impaired insulin sensitivity, obesity, inflammation, and broader metabolic dysfunction in offspring, as reported in individuals with PCOS (20, 22, 42, 44). These metabolic and endocrine disturbances, which often persist into adulthood, may further compromise neurodevelopment and cognitive function through the mechanisms discussed below.

The metabolic disturbances observed in PCOS subjects may also contribute to the cognitive dysfunction associated with this syndrome. IR is a prevalent metabolic disorder among women with PCOS, consistent with the IR observed in our rat model of PCOS (21). As previously reported, hyperinsulinemia and impaired insulin sensitivity adversely affect hippocampal tissue, resulting in cognitive deficits and impaired learning and memory performance (6, 45). Some metabolic disorders, including type 2 diabetes mellitus, have been linked to an increased risk of cognitive dysfunction and dementia, suggesting that the hippocampus is highly sensitive to metabolic stress. Elevated circulating levels of proinflammatory interleukins associated with IR are linked to impaired executive function (46). Cognitive dysfunction has been observed in patients with high levels of inflammatory markers, including women with PCOS. Furthermore, elevated interleukin-18 levels are associated with cognitive decline in patients with AD (47). In addition, IR and hyperinsulinaemia contribute to the accumulation of advanced glycation end products and reactive oxygen species, leading to neurotoxicity and brain damage (48).

Overweight and obesity are frequently reported in individuals with PCOS, and this pattern is consistent with our findings of increased body weights in the PCOS rat model both before puberty and throughout the reproductive period, as demonstrated in the present study and in our previous studies (20, 21). However, as the animals aged, BW differences between the PCOS and control groups were no longer statistically significant. This observation aligns with reports indicating that several clinical and biochemical features of PCOS may attenuate with advancing age (49). Obesity itself is known to exert negative effects on brain structure and function (50). For example, obesity-related alterations in hypothalamic–pituitary–adrenal (HPA) axis activity can lead to increased glucocorticoid secretion, which has been associated with reduced hippocampal volume, impaired memory performance, and heightened susceptibility to mood disorders (51). In addition, obesity can induce neuroinflammation, IR, and mitochondrial dysfunction in the brain (hippocampus), collectively impairing hippocampal neuroplasticity and cognitive function, while also exacerbating neuropathological disorders such as AD (52, 53). These mechanisms collectively suggest that obesity may further contribute to the neurobiological and cognitive alterations observed in PCOS.

Gut microbiota dysbiosis has been reported in women with PCOS, as previously reported in our rat model of PCOS (22). The hippocampus can be negatively affected by GM dysbiosis. GM dysbiosis and altered intestinal permeability can induce immune responses in the brain and promote IR, potentially impairing cognitive functions (7, 54). In rodents, GM dysbiosis was associated with altered hippocampal gene expression, impaired learning, hippocampal atrophy, and reduced levels of neuronal markers (55, 56). Furthermore, another study showed that a high-fat diet, through alterations in the composition of the GM, can affect spatial learning and memory (7).

Hormonal disorders such as hyperandrogenemia and high levels of luteinizing hormone (LH) may negatively affect brain activity and function in PCOS subjects. Hyperandrogenemia, a key biochemical feature of PCOS, was also observed in our rat model of PCOS (20). Women with high levels of testosterone, e.g., women with PCOS, exhibited poorer brain activity and cognitive function, specifically in psychomotor speed and visuospatial learning (8, 18). Androgen excess may be associated with IR, as observed in women with PCOS, and several clinical studies have shown an association between IR and cognitive dysfunction (57, 58), as we reported earlier. Additionally, hyperandrogenism has been reported to be potentially associated with dysregulated adipokine and steroid signaling, contributing to neurodegeneration in the hippocampus (59). In addition to the negative effects of androgen excess on brain activity and function, it has been reported that high levels of LH can also alter brain activity, resulting in cognitive dysfunction (19).

Despite existing evidence of memory dysfunction in some humans and animals with PCOS (9, 10, 38), as well as the observed neuronal damage in hippocampal tissue and impaired spatial learning in middle-aged PCOS rats in the present study, no significant memory impairment was observed in the middle-aged PCOS rat model. The absence of significant memory dysfunction in our middle-aged PCOS rat model may have several explanations. First, androgens may have positive effects on memory. It is important to note that medications and hormones can have a profound impact on the formation of spatial memory. Despite the negative effects of androgens on cognitive function noted above, animal and clinical studies indicate that testosterone can have a positive effect on spatial memory (60). In one experiment, testosterone-treated female rats showed superior long-term spatial memory performance compared to untreated rats, with results comparable to those observed in control male rats (61). Moreover, positive effects of androgens on memory performance have been reported in other studies (62-65). Second, the age of the animals and the timing of behavioral assessment may have influenced the findings. Previous research has demonstrated age-related deficits in spatial memory and associated hippocampal function, although testosterone treatment may improve the cognitive dysfunction associated with the aging process (60). Given that memory dysfunction is widely recognized as an age-related process, it would be prudent to examine memory performance in our rat model of PCOS at an older age in future studies. Third, the behavioral assays employed may have lacked the sensitivity to detect subtle cognitive impairments (memory dysfunction) in early-stage dysfunction, despite hippocampal neural damage and impaired spatial learning. It has been reported that in the initial stages of AD, the hippocampal tissue and its connections with other brain regions become damaged (36). Fourth, learning and memory may involve partly distinct neurobiological mechanisms. Further long-term studies are needed.

In the present study, increased bace1 gene expression was observed in the hippocampal tissue of middle-aged PCOS rats. bace1 is a transmembrane protein and an important β-secretase. bace1 is broadly distributed throughout the brain, with pronounced expression in neurons, oligodendrocytes, and astrocytes, and is especially abundant across diverse neuronal subtypes. Numerous studies have indicated that bace1 is essential for amyloid β production and is implicated in AD pathogenesis (66, 67). Studies in transgenic mice suggest that bace1 may be the major β-secretase involved in plaque formation in the brain (68-71). One study showed that bace1 gene expression was 14-fold higher in the blood of the AD group than in controls (72). Another study demonstrated that bace1 activity increases in the brain tissue and cerebrospinal fluid of subjects with mild cognitive impairment and probable AD (73). Upregulation of bace1 in brain tissues and high plasma levels of bace1 have been observed in patients with AD (73-75).

Decreased mapt gene expression was observed in the hippocampal tissue of our middle-aged PCOS rat model; this finding is consistent with previous studies (76, 77). A previous study showed that mapt gene expression was lower than that in controls in a rat ischemic model of AD (76). Additionally, in AD patients, higher tau protein but lower total mRNA levels of tau have been reported (77). In one study of women with PCOS, mapt protein levels were comparable between PCOS subjects and their controls (78).

### 5.1. Strengths and Limitations

A key strength of this study lies in its novelty, as it represents one of the earliest investigations into the impact of PCOS history on spatial learning, memory, and hippocampal tissue (histopathology and the expression of genes involved in AD pathogenesis, including bace1, mapt, app, nes, and dcx) in rats later in life. Other strengths of the present study include the induction of PCOS following prenatal androgen excess exposure and the age of the rats at the time of investigation. Prenatally induced PCOS animal models are considered superior to postnatally induced animal models for studying this syndrome and associated disorders, as they may result in permanent changes that more closely resemble the development and conditions observed in human PCOS. It is postulated that in postnatally induced animal models, PCOS features and associated disorders may be transient and dependent on the timing of the intervention, potentially reverting to normal after cessation of treatment. Furthermore, our prenatal androgen-induced rat model of PCOS was evaluated at a later stage in life (12 months of age), in contrast to most postnatally induced PCOS models, in which animals are assessed immediately after treatment.

Several limitations should be acknowledged in the present study. The limited number of similar animal studies constrains the interpretation of our results. The lack of a significant difference in the expression of app, nes, and dcx genes in the hippocampal tissue of PCOS rats compared with controls could be attributed to the age of the animals and the stage of neurocognitive dysfunction (AD). As the pathological processes underlying AD are known to evolve over time, it is possible that the observed gene expression patterns may differ at various stages of the disease. qRT-PCR normalization was performed using a single reference gene (ywhaz), although three candidate reference genes were initially evaluated. This study did not include immunohistochemical analysis to assess neuronal damage, neuroinflammation, and synaptic integrity (e.g., caspase-3, glial fibrillary acidic protein, ionized calcium-binding adaptor molecule 1, and synaptophysin). The correlation between obesity-related measures and the observed cognitive, molecular, and histopathological outcomes was not directly evaluated in the present study. Another limitation of the present study is the exclusive evaluation of ovaries at 12 months of age. While characterizing ovarian status is essential, ovarian tissue in rats at this life stage typically exhibits advanced, age-related atrophic changes, and the ovaries were unsuitable for evaluation. These physiological shifts impede a reliable assessment of PCOS-specific ovarian lesions and preclude the establishment of a robust correlation between ovarian pathology and hippocampal alterations.

Longitudinal investigations tracking gene expression and histological changes in the hippocampus across different age groups and disease stages would provide a more comprehensive understanding of the relationship between PCOS and AD risk.

### 5.2. Conclusions

In conclusion, our middle-aged PCOS rat model exhibited hippocampal histopathological damage and impaired spatial learning, whereas spatial memory remained preserved, potentially reflecting androgen-mediated neuroprotection. In addition, alterations in the expression of some genes involved in AD pathogenesis were observed in this middle-aged PCOS rat model. These findings suggest that PCOS may serve as a risk factor for cognitive dysfunction and AD in later life. This rat model of PCOS may be considered a robust framework for investigating the cellular and molecular mechanisms underlying PCOS-associated cognitive decline and for evaluating future therapeutic interventions.

## Data Availability

All relevant data presented in this study are included in the article. Any other data that support the findings discussed here are available from the corresponding author on reasonable request.
